# Supplementary material for: Candidate Gene Identification and Transcriptome Analysis of Tomato male sterile-30 and Functional Marker Development for ms-30 and Its Alleles, ms-33, 7B-1, and stamenless-2
Source: Int J Mol Sci. 2024 Mar 15;25(6):3331. doi: 10.3390/ijms25063331 (PMC10970333; doi:10.3390/ijms25063331)
Supplement: Supplementary file 1 [file ijms-25-03331-s001.zip › Supplementary Material A ms-30 supplementary material Figures S1~S8.pdf]

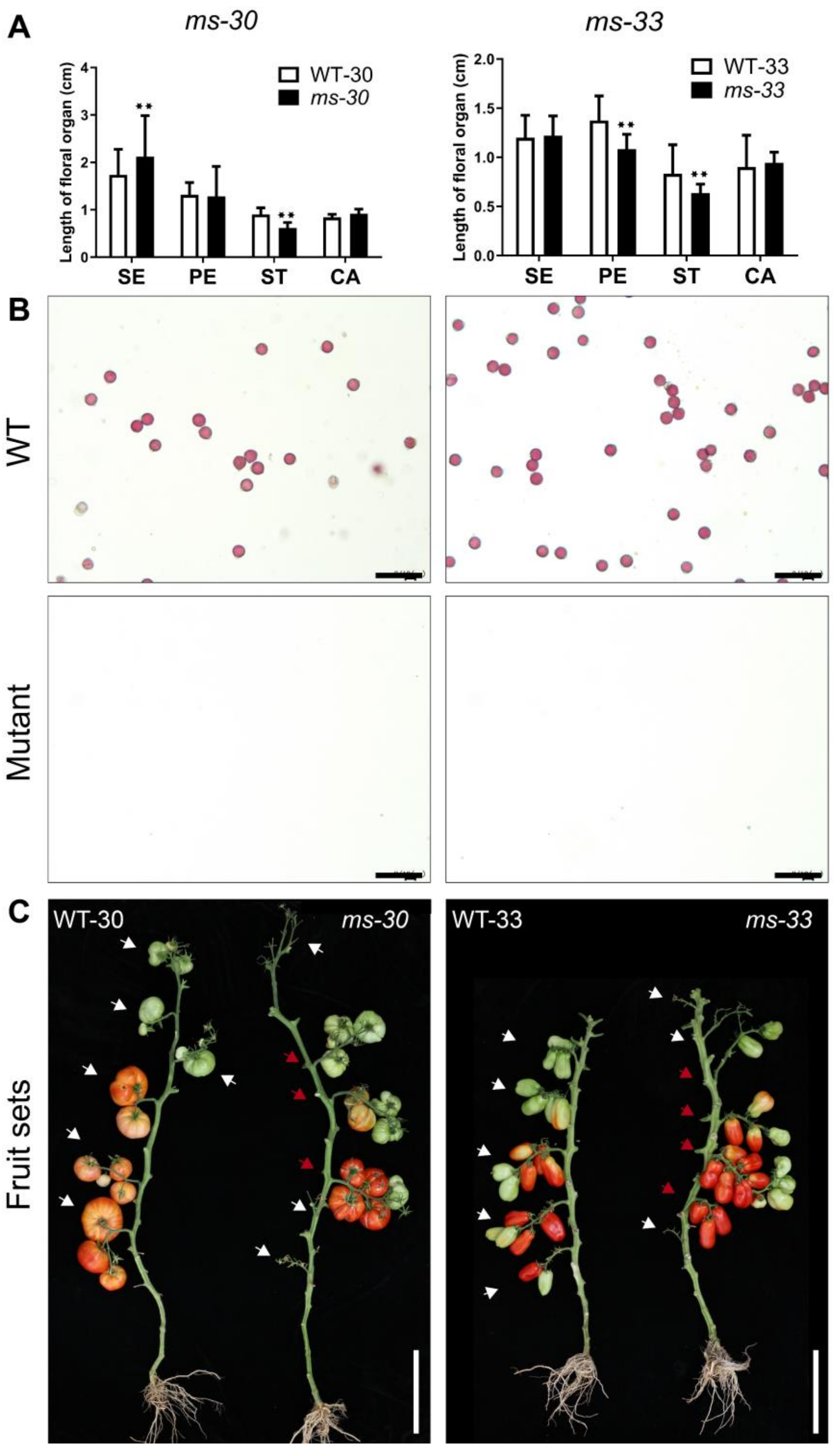

Figure S1. Length of floral organs, phenotypes of pollens and fruit sets in *ms-30* and *ms-33*. (A) Length of floral organs at the anthesis. Asterisks indicate a significant difference ($p < 0.05$) between the WT and mutant plants; (B) Analysis of pollen viability by staining the pollens with acetocarmine. Scale bars, 1 mm; (C) Fruit sets of *ms-30* and *ms-33*. White arrows indicate self-cross, and red arrows indicate out-cross. Scale bars, 20 cm.

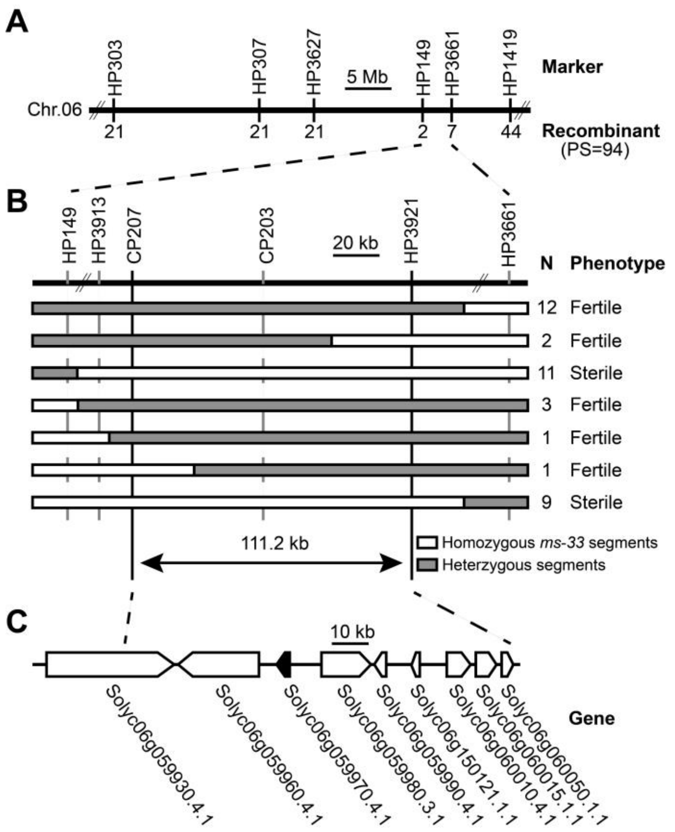

Figure S2. Fine-mapping of *ms-33*. (A) Preliminary mapping of *ms-33*. PS indicates population size. (B) Fine mapping of *ms-33*. N indicates the number of recombinants; 39 recombinants selected from 861 plants of the F_2 population. (C) ITAG4.0 annotated genes of *ms-33*. Arrows indicate the direction of transcription, and the solid arrow presents the most likely candidate gene for *ms-33*.

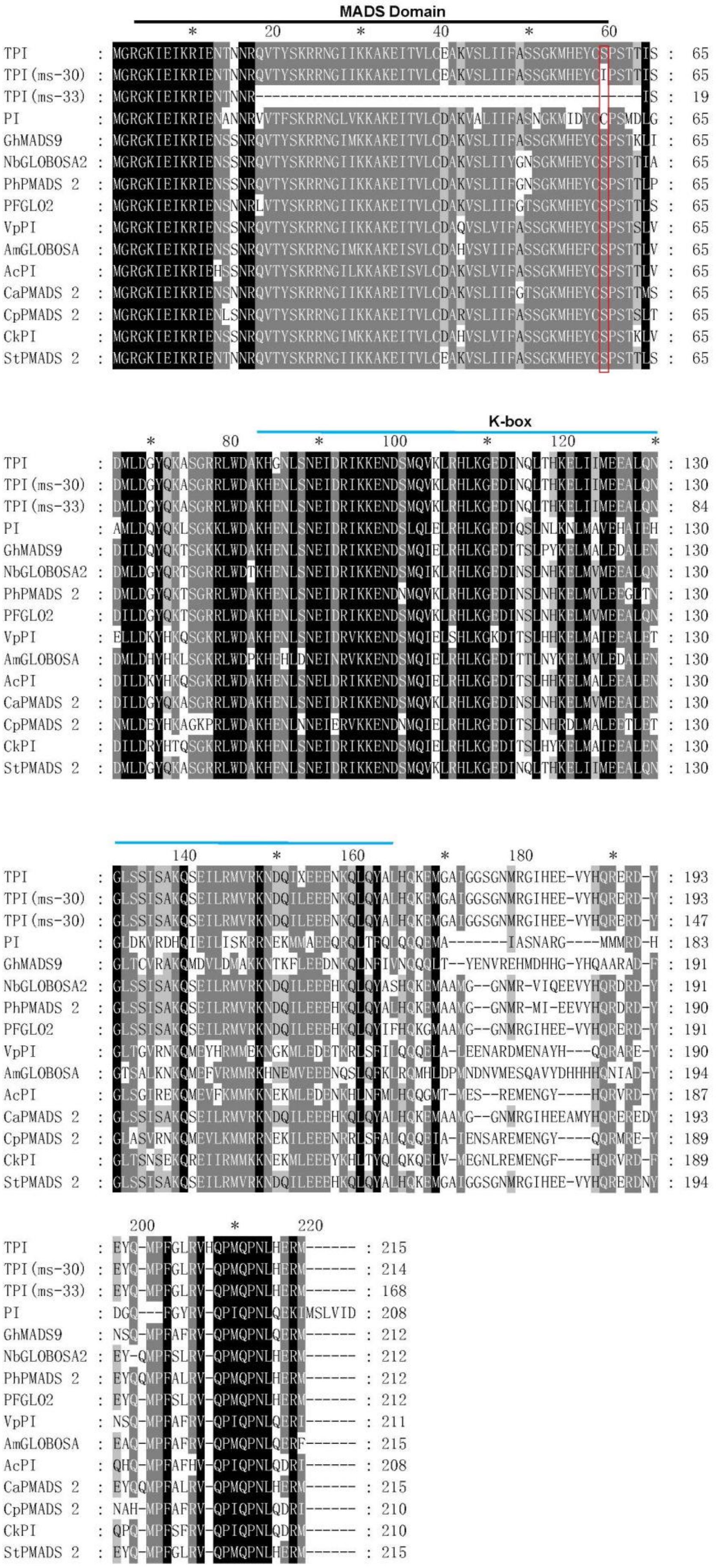
MADS Domain
* 20 * 40 * 60
TPI : MGRGKIEIKRIENTNNRQVTYSKRRNGIIKKAKEITVLCEAKVSLIIFASSGKMHEYCSPSTTIS : 65
TPI(ms-30) : MGRGKIEIKRIENTNNRQVTYSKRRNGIIKKAKEITVLCEAKVSLIIFASSGKMHEYCIPSTTIS : 65
TPI(ms-33) : MGRGKIEIKRIENTNNR--IS : 19
PI : MGRGKIEIKRIENANNRVVTFSKRRNGLVKKAKEITVLCDAKVALIIFASNGKMIDYCCPSMDLG : 65
GhMADS9 : MGRGKIEIKRIENSSNRQVTYSKRRNGIMKKAKEITVLCDAKVSLIIFASSGKMHEYCSPSTKLI : 65
NbGLOBOSA2 : MGRGKIEIKRIENSSNRQVTYSKRRNGIIKKAKEITVLCDAKVSLIIYGNSGKMHEYCSPSTTIA : 65
PhPMADS 2 : MGRGKIEIKRIENSSNRQVTYSKRRNGIIKKAKEITVLCDAKVSLIIFGNSGKMHEYCSPSTTLP : 65
PFGLO2 : MGRGKIEIKRIENSNNRLVTYSKRRNGIIKKAKEITVLCDAKVSLIIFGTSGKMHEYCSPSTTLS : 65
VpPI : MGRGKIEIKRIENSSNRQVTYSKRRNGIIKKAKEITVLCDAQVSLVIFASSGKMHEYCSPSTSLV : 65
AmGLOBOSA : MGRGKIEIKRIENSSNRQVTYSKRRNGIMKKAKEISVLCDAHVSVIIFASSGKMHEFCSPSTTLV : 65
AcPI : MGRGKIEIKRIEHSSNRQVTYSKRRNGILKKAKEISVLCDAKVSLVIFASSGKMHEYCSPSTTLV : 65
CaPMADS 2 : MGRGKIEIKRIENSNNRQVTYSKRRNGIIKKAREITVLCDAKVSLIIFGTSGKMHEYCSPSTTMS : 65
CpPMADS 2 : MGRGKIEIKRIENLSNRQVTYSKRRNGIIKKAKEITVLCDARVSLIIFASSGKMHEYCSPSTSLT : 65
CkPI : MGRGKIEIKRIENSSNRQVTYSKRRNGIMKKAKEITVLCDAHVSLVIFASSGKMHEYCSPSTKLV : 65
StPMADS 2 : MGRGKIEIKRIENTNNRQVTYSKRRNGIIKKAKEITVLCEAKVSLIIFASSGKMHEYCSPSTTLS : 65
K-box
* 80 * 100 * 120 *
TPI : DMLDGYQKASGRRLWDAKHGNLSNEIDRIKKENDSMQVKLRHLKGEDINQLTHKELIIMEEALQN : 130
TPI(ms-30) : DMLDGYQKASGRRLWDAKHENLSNEIDRIKKENDSMQVKLRHLKGEDINQLTHKELIIMEEALQN : 130
TPI(ms-33) : DMLDGYQKASGRRLWDAKHENLSNEIDRIKKENDSMQVKLRHLKGEDINQLTHKELIIMEEALQN : 84
PI : AMLDQYQKLSGKKLWDAKHENLSNEIDRIKKENDSLQLELRHLKGEDIQSLNLKNLMAVEHAIEH : 130
GhMADS9 : DILDQYQKTSGKKLWDAKHENLSNEIDRIKKENDSMQIELRHLKGEDITSLPYKELMALEDALEN : 130
NbGLOBOSA2 : DMLDGYQRTSGRRLWDTKHENLSNEIDRIKKENDSMQIKLRHLKGEDINSLNHKELMVMEEALQN : 130
PhPMADS 2 : DMLDGYQKTSGRRLWDAKHENLSNEIDRIKKENDNMQVKLRHLKGEDINSLNHKELMVLEEGLTN : 130
PFGLO2 : DILDGYQKTSGRRLWDAKHENLSNEIDRIKKENDSMQVKLRHLKGEDINSLNHKELMVMEEALQN : 130
VpPI : ELLDKYHKQSGKRLWDAKHENLSNEIDRVKKENDSMQIELSHLKGKDITSLHHKELMAIEEALET : 130
AmGLOBOSA : DMLDHYHKLSGKRLWDPKHEHLDNEINRVKKENDSMQIELRHLKGEDITTLNYKELMVLEDALEN : 130
AcPI : DILDKYHKQSGKRLWDAKHENLSNELDRIKKENDSMQIELRHLKGEDITSLHHKELMALEEALEN : 130
CaPMADS 2 : DILDGYQKASGRRLWDAKHENLSNEIDRIKKENDSMQVKLRHLKGEDINSLNHKELMVMEEALEN : 130
CpPMADS 2 : NMLDEYHKAGKPRLWDAKHENLNNEIERVKKENDNMQIELRHLRGEDITSLNHRDLMALEETLET : 130
CkPI : DILDRYHTQSGKRLWDAKHENLSNEIDRIKKENDSMQIELRHLKGEDITSLHYKELMAIEEALEN : 130
StPMADS 2 : DMLDGYQKASGRRLWDAKHENLSNEIDRIKKENDSMQVKLRHLKGEDINQLTHKELIIMEEALQN : 130
140 * 160 * 180 *
TPI : GLSSISAKQSEILRMVRKNDQIXEEENKQLQYALHQKEMGAIGGSGNMRGIHEE-VYHQRERD-Y : 193
TPI(ms-30) : GLSSISAKQSEILRMVRKNDQILEEENKQLQYALHQKEMGAIGGSGNMRGIHEE-VYHQRERD-Y : 193
TPI(ms-30) : GLSSISAKQSEILRMVRKNDQILEEENKQLQYALHQKEMGAIGGSGNMRGIHEE-VYHQRERD-Y : 147
PI : GLDKVRDHQIEILISKRRNEKMMAEEQRQLTEQLQQQEMA-------IASNARG----MMMRD-H : 183
GhMADS9 : GLTCVRAKQMDVLDMAKKNTKFLEEDNKQLNFIVNQQQLT--YENVREHMDHHG-YHQAARAD-F : 191
NbGLOBOSA2 : GLSSISAKQSEILRMVRKNDQILEEEHKQLQYASHQKEMAAMG--GNMR-VIQEEVYHQRDRD-Y : 191
PhPMADS 2 : GLSSISAKQSEILRMVRKNDQILEEEHKQLQYALHQKEMAAMG--GNMR-MI-EEVYHQRDRD-Y : 190
PFGLO2 : GLSSISAKQSEILRMVRKNDQILEEEHKQLQYIFHQKGMAAMG--GNMRGIHEE-VYHQRERD-Y : 191
VpPI : GLTGVRNKQMEYHRMMEKNGKMLEDETKRLSFILQQQELA-LEENARDMENAYH---QQRARE-Y : 190
AmGLOBOSA : GTSALKNKQMEFVRMMRKHNEMVEEENQSLQFKLRQMHLDPMNDNVMESQAVYDHHHHQNIAD-Y : 194
AcPI : GLSGIREKQMEVFKMMKKNEKMLEDENKHLNFMLHQQGMT-MES--REMENGY----HQRVRD-Y : 187
CaPMADS 2 : GLSSISAKQSEILRMVRKNDQILEEEHKQLQYALHQKEMAAMG--GNMRGIHEEAMYHQREREDY : 193
CpPMADS 2 : GLASVRNKQMEVLKMMRRNEKILEEENRRLSFALQQQELA-IENSAREMENGY----QQRMRE-Y : 189
CkPI : GLTSNSEKQREIIRMMKKNEKMLEEEYKHLTYQLQKQELV-VEGNLREMENGF----HQRVRD-F : 189
StPMADS 2 : GLSSISAKQSEILRMVRKNDQILEEEHKQLQYALHQKEMGAIGGSGNMRGIHEE-VYHQRERDNY : 194
200 * 220
TPI : EYQ-MPFGLRVHQPMQPNLHERM------ : 215
TPI(ms-30) : EYQ-MPFGLRV-QPMQPNLHERM------ : 214
TPI(ms-33) : EYQ-MPFGLRV-QPMQPNLHERM------ : 168
PI : DGQ---FGYRV-QPIQPNLQEKIMSLVID : 208
GhMADS9 : NSQ-MPFAFRV-QPMQPNLQERM------ : 212
NbGLOBOSA2 : EY-QMPFSLRV-QPMQPNLHERM------ : 212
PhPMADS 2 : EYQQMPFALRV-QPMQPNLHERM------ : 212
PFGLO2 : EYQ-MPFSLRV-QPMQPNLHERM------ : 212
VpPI : NSQ-MPFAFRV-QPIQPNLQERI------ : 211
AmGLOBOSA : EAQ-MPFAFRV-QPMQPNLQERF------ : 215
AcPI : QHQ-MPFAFHV-QPIQPNLQDRI------ : 208
CaPMADS 2 : EYQQMPFALRV-QPMQPNLHERM------ : 215
CpPMADS 2 : NAH-MPFAFRV-QPIQPNLQDRI------ : 210
CkPI : QPQ-MPFSFRV-QPIQPNLQDRI------ : 210
StPMADS 2 : EYQ-MPFGLRV-QPMQPNLHERM------ : 215

Figure S3. Multiple alignment of TPI proteins based on their full-length sequences. Entire protein sequences were aligned using ClustalW in MEGA X (Kumar et al., 2018) with default settings, and conserved amino acids are shaded using GeneDoc (Nicholas and Nicholas, 1997). The MADS domain and K-box are marked above the alignment. TPI presents the TPI protein in WT-30 and WT-33. TPI (*ms-30*) indicates the TPI protein in *ms-30* mutant and TPI (*ms-33*) indicates the protein in the ms-33 mutant. The sequences of these proteins were downloaded from NCBI GenBank by using the following accession numbers: PI (AAD51988.1), GhMADS9 (XP_016742051.1), NbGLOBOSA2 (AWD93715.1), PhPMADS 2 (Q07474.1), PFGLO2 (AGN54426.1), VpPI (APQ46145.1), AmGLOBOSA (Q03378.1), AcPI (ADU15475.1), CaPMADS 2 (NP_001311952.1), CpPMADS 2 (XP_021902590.1), CkPI (AGA61757.1), and StPMADS 2 (XP_006365936.1).

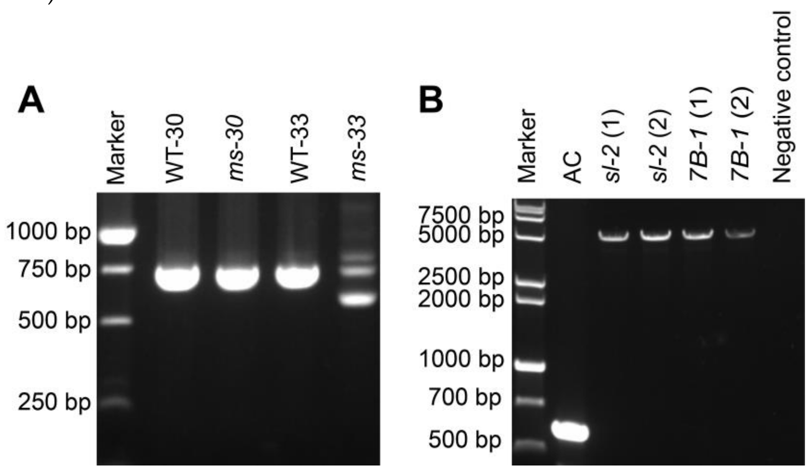

Figure S4. Electrophoresis detection of alternatively spliced transcripts in *ms-33* and the retrotransposon insertion in *7B-1* and *sl-2*. (A) Agarose gel electrophoresis of *TPI* CDS amplified from the flower cDNA of WT or mutant plants of *ms-30* or *ms-33* using the primer pair TPICDS. (B) Agarose gel electrophoresis of PCR fragments amplified from the WT genome DNA, two plant genome DNAs of the *sl-2* mutant, and two plant genome DNAs of *7B-1* using the primer pair LeGLO2-6.

GGTTTTCTCGGGGATTAGTATGAAAATTTACAGGAAAATAAGTTTCATGCGAATTTTCATACTAATTTCTACGAAAATCTCCATATTATTCATAGTTTTCTTC
GAGTGATAGTGACAAATTAAAAATAGATGTTTGGTTAACTATTTTGTCTCTTCAAATTGTATGTTTTTGGATTCCTAAAGCACCAAAAGGAGATGGGAGCCAT
TGGTGGAAGTGGAAATATGAGAGGAATTCATGAAGAAGTGTATCTGTTGTGCGGAATTTGAGATAATACGAGAAAATATAAACGCGAAAAATAAGACAACAGA
TTTACGTGGTTCACCAACAAATTGGCTACGTCCACGGGAAGAGAGGGAGCAGTTTTATTATGGAGAGGCAAAAACAGAATTACAGAATAGGGTTTCCCATAGC
GTCTATATATAGTGCTAAGCTACGCCCTAACAGGCTTGGGCCCAACATACAGAATCAACAGAAAATTAAGGGCCCAATACAACAACATTGTATACCGTCGGCC
CGGGGGCGTCTCCGCCCCCCCGGACCCCCAGGCCAGGGGGCGCGTCGCCCCCCTGGACCCCCCGACTCGCTGACCGGGCAGCGAGACCCCCGTCCTTTCTGTT
TGTAGCGGGTCCGATTCAAGGCATTCAACAAATCTCCACCTTGACTTGAATTCTCCGAACAGATTCTTCAGACGCACTATGATAGTGCCAAGCCTCCCCCTCT
TCCTCAGAGTTGCCCCGCAGGGCAATTAACAGCTTCTGATGTTGAGCAAGTCCAAACAGTGTTGAAACTTGCTCTGTGGAACCGGCTTTGTGAACATATCAGC
AGGATTATCAGCAGTTCCTACTTTCTTCACCTTGATTCTCTTCTCACTTCTTAGAAAATGATACCTTACGTCAATATGCTTGGTTCTCTCATGATGGACTTGA
TCCTTGGCTAGACAAATTGCGCTCAAACTGTCACAATACACCGTAGCCTGATCATGATGCAGACCAAGATCACTAACCAGCCCTTTCAACCAAATCCCTTCTT
TTGCAGCCTCTGTCAAGGCCATGTACTCCGCTTCCGTAGTAGACAAAGTCACTGTAGGTTGCAAAGTTGCCTTCCAACTGACGACAGATCCTCCAAGGGTAAA
CACATAGCCAGTCATCGATCTTCTTGTGTCAACATCTCCAGCATAGTCTGAATCAGAATAGCCAGTAACCAAGCACTGAGTATCACCTCCATAAATGAGACCA
ACGTCAGATGTACCTCTAAGGTACCGGAAAATTCTCTTCACAGCCTGCCAATGTTCTCTCCCTGGTTGTCCCATGAATCTGCTCACTACACTGACTGCATGTG
CTAAATCTGGCCTTGTACAGACCATAGCATACATCAAACTTCCTACGGCACTGGCATAAGGGACTCGTGACATATACTCCTTCTCTTCTTCTGACTGTGGAGC
GAACATGGCAGTGAGATGGATATTGGCAGCACTGGGGGTATCAATGGGCTTAGATGAAGACATGCCAAACCTCGCCAAGACCTTCTGAATGTAGCTTCTCTGT
GACAAGAAAAGTTTCCTTCTCTCTCTGTCTCTAATGATCTCCATCCCTAAAATCTTCCGAGCGGCTCCCAGATCCTTCATCTCAAACTCAGCACTAAGTAAAC
CCTTCAGCTTCTGAATGTCATACTTCTTCTTTGCAGCTATCAACATATCATCTACATAAAGCACCAGATAGATGAATGAATCATCATTGAGCCTATTGTAGTA
GACACAACAATCATATGAGCTCCGAGTATAGCCCAACTTCACCATATAGCTGTCAAACCTTTTATACCACTGCCTTGGAGACTGCTTAAGTCCATATAAGGAC
TTCTTCAACTTGCAGACGTGATTTTCCTTCCCTGGAACTTGGAAACCATCCGGCTGAGTCATGTATATCTCTTCCTCCAACTCTCCATGTAGAAACGCTGTCT
TCACATCAAGTTGTTCAAGCTCCAGATTCTGATGTGCAACTATCGCTAGTAACACTCGGATGGAAGTATGTCTGACCACTGGTGAGAAGATCTCATTGTAGTC
CACTCCCTCTCTTTGGTTGAAACCTCTGGCAACAACCCTGGCTTTATACTTGACTCCTTCTGCTGGTGATATCCCTTCCTTCTTCTTGAAAACCCATTTGCAA
GTAATAATCTTTCTCCCCGAAGGCTGTATGACCAGATCCCATGTCTGATTCTTGTGTAGGGACTCCATCTCATCTCCCATAGCGGCAAACCATTTTTCAGAAT
CAGAACTTAAAATGGCTTCTTTGTAAGTAGACGGCTCAGATGTATCTACCTCTTCAGCAACCTGCAGTGCATAACCCACCATGTCCTCAAAACCATACCTCGT
AGGTGGCCGAACTCCAACCCTCCTTGGCCGATCTTGAGCTATACTCTGATGGATATCTGATGGCATAGATTCTGGAATATCAGTTTCAGTCTGTGGCTCTTGA
TCCTCCTCTTCAGGTTCCTTTAAATCGCTCTCGTTCTGAATGACTTGAAACTCCACCTGTTTGTCAAGACTCCCAGTTTCTGACGTAGTTGTAGGCTTCACAA
TGGTTCTAAGCAGAGGACTTTCATCAAAGACAACGTTCCTGCTCATAATAACCCTCTTTTCTGCTGGAGACCAGATTCTGAAACCTTTCACTCCATCTCCGTA
GCCCACAAATACTCCCTTTTTAGCTCTTGGTTCTAACTTACCTTCACTGACGTGATAGTAAGCCGTACAACCAAAAGCTTTCAGATTTGAATAATCAGCAGCT
TTTCCAGACCACATCTCCATAGGTGTCTTGCACTGTATACCTGTATGTGGTCCGCGGTTAATCAAGTAGCAAGCTGTACTAACCGCTTCTGCCCAGAATCTTC
TATCTAGCCCAGCATTAGAGAGCATGCACCTTGCTCTCTCCAGAAGTGTTTGATTCATCCGCTCAGCTACACCGTTCTGCTGTGGTGTATTTCTGACTGTGCG
ATGTCGAGCAATCCCTTCATCCTTACAGAATTGATCAAATTCAGACCAACAGAATTCCAGCCCATTATCAGTTCGCAACCTCTTGATCTTCTTCCCTGTTTGA
TTTTCCATCAAAATTTTCCACTCCTTGAACTTCTGGAAGGCTTCACTTTTATGCTTCATCATGTACACCCAAGTCATCCTTGAGTAGTCATCAATAATGGACA
CAAAAAATCTGCAGCCTCCCAAAGACTCAACACGGCATGGACCCCAGCAATCAGAATGGATATAATCAAGTGTGCCTTTTGTTCTATGAATGGCCTTTGGAAA
CTTGTTGCGATGTAGTTTTCCAAAAACACAATGTTCACAAAACTCTAGGCTCTTAACCTTATGACCAGCAAGTAAATCCTCCTTTGACAGAATTTGCATCCCT
CTTTCACCCATATGACCAAGTCTTATGTGCCATAACTTAGTCATATCCTTCTGGTGAAATTCTGACGATGCAACATGGGCTGAACCTGTAACCGTGGAACCTT
GTAGAAAATACAAAGTACCACGCATGACACCTTTCAGAATCAAATTTGAACCCTTCCAGACCCGCAAGACTCCATCTTTTCCCGACCAGCTGAATCCCTTGCT
GTCCAAAAAGACTGAGAGATATCAGATTTTTCGTCATCAATGGAACGTGCCTGACCTCGTTCAATGTGCAGAAGCTACCGTCATGTGTCCTTATCTTGATCGA
GCCTGTCCCAACCACCTTGCAGACAGAACTGTTGGCCATCGAGATGCTGCCTCCGTCTACCTGCTCATAAGTCGTGAACCACTCTCTCCTAGGACAGATGTGA
TAGGATGCCCCAGAATCAAGAACCCACACATCTGAATGATGAGTGTGCTCATCCGCAACTAGGGCAATATCTTCTTCAGAATTGGTGTCTTCTTCAGCAACAG
CAGCAGACACTGATTGTTTTTCCGATTGCTTCTTCTTCTTCGGACAATCAAATTTCCAATGTCCCTTCTCCTTGCAGTAATTACAAACATCATCCGGCTTTGC
ACCCTTCGACATCGGCTTATTTTTCTTTCCGCCGTTTTTCCTTCCCTTTCTGCTACTGGTGAACAGACCGGAAGGCTGTATGTCCGTACTTGTGCCGTTAGCC
TTATGCCGTAATTCCCTGCTATGAAGGGCTGATCTGACTTCTTCCAGTGACACAGTATCTTTCCCAACAATGAACGATTGAACAAAATTCTCAAACGACATTG
GGAGAGATACTAACAGAATCAGGGCAGCATCTTCATCCTCGATCTTCACATCGATATTACGCAATTCTAATAACAAAGTATTCAATTGCTCTAAGTGTTCCCT
GAGTTGTGTACCTTCAGCCATTCGTAAACCGAATAGACGTTGTTTCAGAAGCAGCTTGTTGGTTAGAGATTTTGTCATGTACAAACTCTCCAGCTTCAACCAC
AGACCAGCAGCAGTCTCTTCATCCGAGACCTCCGTGATGACGTCATCCGCGAGACACAGCATGATCGTCGAGTGCGCCTTTTCCTCCAGAATCGCCATCTCAG
GAGTAACGACGGCGTTCTTGTCTTTCGACAACGGCGCCCAGAAGCCTTGCTGTTTCAACAAGGCCCGCATCTTGATCTGCCATAAACTGAAACTGTTCCTCCC
TGTGAATTTGTCGATTTTCACGTTCAAAGCAGACATCTCGAATTCTCCAAGAACACCGATTAACCGAGAGGCTCTGATACCAGTTTGTTGTGCGGAATTTGAG
ATAATACGAGAAAATATAAACGCGAAAAATAAGACAACAGATTTACGTGGTTCACCAACAAATTGGCTACGTCCACGGGAAGAGAGGGAGCAGTTTTATTATG
GAGAGGCAAAAACAGAATTACAGAATAGGGTTTCCCATAGCGTCTATATATAGTGCTAAGCTACGCCCTAACAGGCTTGGGCCCAACATACAGAATCAACAGA
AAATTAAGGGCCCAATACAACAACATTGTATACCGTCGGCCCGGGGGCGTCTCCGCCCCCCCGGACCCCCAGGCCAGGGGGCGCGTCGCCCCCCTGGACCCCC
CGACTCGCTGACCGGGCAGCGAGACCCCCGTCCTTTCTGTTGTAGCGGGTCCGATTCAAGGCATTCAACAGTATCATCAAAGAGAAAGGGATTATGAGTACCA
AATGCCATTTGGCCTACGAGTTCAGCCAATGCAGCCAAATCTACATGAAAGAATGTAAAATTATATTAATTCCCACTTTAAATTAAGAACTCTAATAATTTTA
ATTTGGGTTTCTACTATAGCTAGAAACACTAATTAATCAATTCTAGTTATATGTAATGAGTATTAGCTAAGACTTTGTTTCATGTTGGAATTATTACCTAATT
TTATGGGCTCTTCCCATGGTTTTAATTAAGTACTGAAATGTAGTGACATATTTATATGAATTGTTCTACTTACCTAGCTACTACATTTTTTCAATTTGATTT
TGGTCTAGATGGATTCTGAAAAC

Figure S5. Sequence of the long terminal repeat retrotransposon inserted in the *TPI* gene in *7B-1* and *sl-2* mutants. Green letters represent the genome sequence of the *TPI* gene, whereas gray letters represent the direct repeat sequence of the retrotransposon. Red letters represent the target site duplication (TSD) sequence. The fragment was amplified using the primer pair LeGLO2-6 (Pucci et al., 2017) from *7B-1* and *sl-2* mutants.

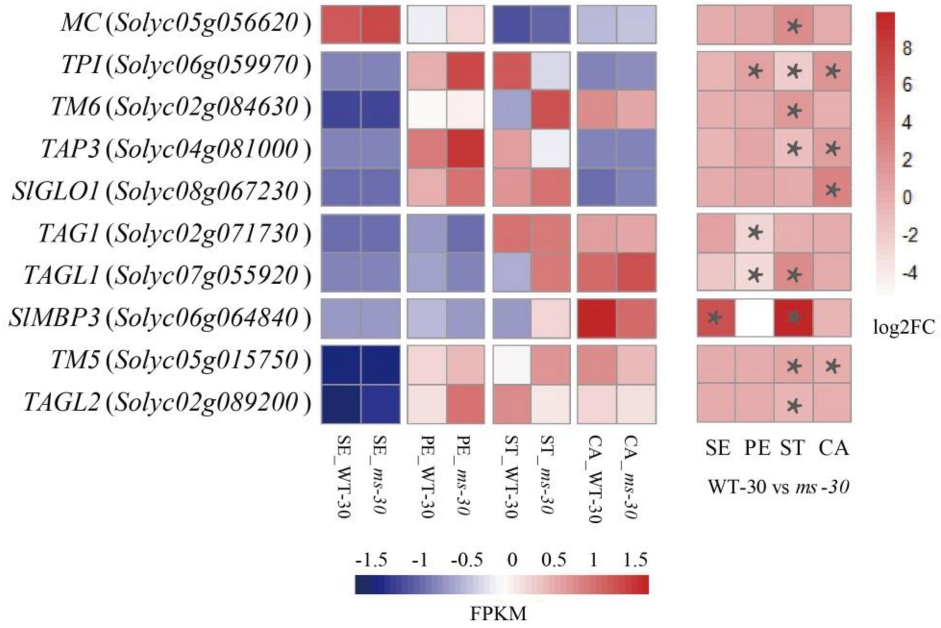

Figure S6. Heatmap of tomato ABCDE model genes between WT-30 and *ms-30*. *MC* is A-class gene; *TPI*, *TM6*, *TAP3*, and *SlGLO1* are B-class genes; *TAG1* and *TAGL1* are C-class genes; *SlMBP3* is a D-class gene; and *TAGL2* and *TM5* are E-class genes. The heatmap of FPKM is shown on the left. The heatmap of log2FC is shown on the right. Asterisks indicate a significant difference (*, $p < 0.05$) between WT-30 and *ms-30*. SE, Sepals; PE, Petals; ST, Stamens; and CA, Carpels.

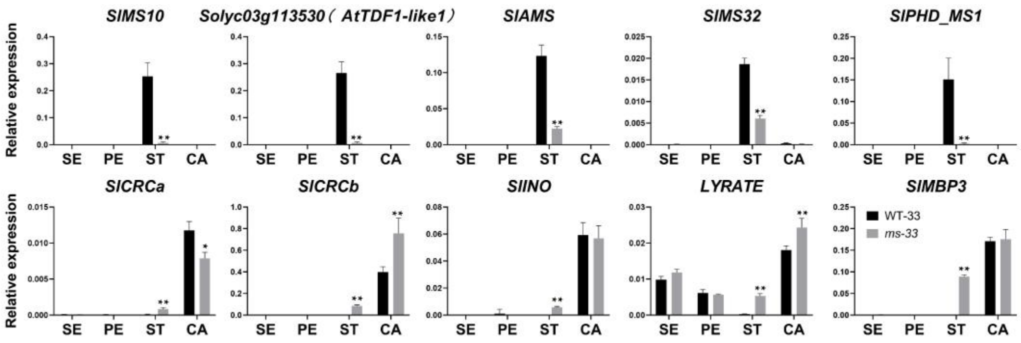

Figure S7. Expression patterns of stamen and carpel development-related genes in the flowers of WT-33 and *ms-33*. Asterisks indicate a significant difference (*, $p < 0.05$; **, $p < 0.01$) between WT-33 and *ms-33*. SE, Sepals; PE, Petals; ST, Stamens; and CA, Carpels

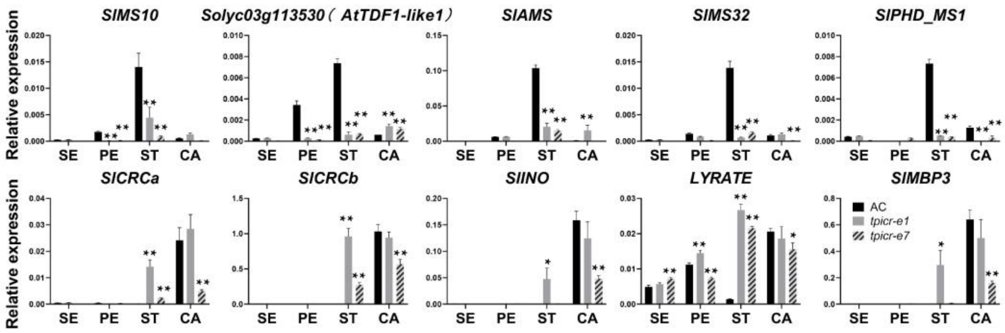

Figure S8. Expression patterns of stamen and carpel development-related genes in the flowers of AC and the *TPI* gene-edited mutants *tpicr-e1* and *tpicr-e7*. Asterisks indicate a significant difference (*, $p < 0.05$; **, $p < 0.01$) between AC and *tpicr-e1, tpicr-e7*. SE, Sepals; PE, Petals; ST, Stamens; and CA, Carpels

References

Kumar, S., Stecher, G., Li, M., Knyaz, C., Tamura, K., 2018. MEGA X: molecular evolutionary genetics analysis across computing platforms. Mol Biol Evol, 35: 1547-1549.

Nicholas, K. B., Nicholas, H. B., 1997. GeneDoc: A tool for editing and annotating multiple sequence alignments. Ver. 2.7.000. 1996. Distributed by the author.
